# Licensing policy and platform models of telemedicine: A multi-case study from China

**DOI:** 10.3389/fpubh.2023.1108621

**Published:** 2023-02-02

**Authors:** Zhong Wang, Rui Xu, Yan Liu, Yiming Li

**Affiliations:** ^1^School of Economics, Guangdong University of Technology, Guangzhou, China; ^2^Key Laboratory of Digital Economy and Data Governance, Guangdong University of Technology, Guangzhou, China; ^3^School of Information Management, Wuhan University, Wuhan, China; ^4^China Center for Information Industry Development, Beijing, China

**Keywords:** telemedicine, platform model, licensing policy, digital health, platform economy, multi-case study, policy implementation, China

## Abstract

**Introduction:**

As a form of platform economy, telemedicine is not growing as fast as other digital platforms. The existing literature seldom pays attention to how licensing policy affects the development of telemedicine platform models.

**Methods:**

This paper uses the method of multi-case study and the theory of policy implementation as mutual adaptation to research the influence mechanism of telemedicine platform licensing policy on the platform model in China.

**Results:**

The findings of the current study are as follows: (1) three models can be classified in accordance with different platform providers in China: medical institution platform, Internet company platform and local government platform; (2) bargaining power, reputation mechanism and resource specificity are important dimensions in the analysis of platform models; (3) as an implementer in the process of licensing policy, the platform provider can not only directly determine the establishment and formation of platform model but also indirectly affect the sustainable development of platform model by affecting the supplier and the demander of platform; and (4) The impact between licensing policy and platform model is dynamic and bidirectional, mainly exerted *via* administrative orders, market-oriented mechanism and medical insurance.

**Conclusions:**

The research enlightens practical exploration in telemedicine and enriches the theoretical innovation in platform.

## Introduction

Telemedicine (also called telehealth) has developed rapidly in China, influenced by various factors such as the gradually aggravated population aging, the insufficient supply of high-quality medical resources, and the rapid development of digital technology (1, 2). The process has been further accelerated by the outbreak of COVID-19 (3–5). At present, telemedicine in a broad sense includes at least the dimensions of diagnosis and treatment, nursing, and medical circulation (6, 7). This study mainly focuses on narrow telemedicine, that is, Internet diagnosis and treatment services. Telemedicine is a classic form of the platform economy. The supplier is the doctor, including specialists and general practitioners (GPs); the demander is the patient; and the mobile application or desktop website is the platform. The application gathers the suppliers and the demanders, which has a scale effect and a network effect and also has cross-group positive externalities (8). As a digital platform, after breaking through a certain user scale, its development will be accelerated by the three effects mentioned earlier (9–12). However, as a highly regulated industry, the establishment and development of the platform are heavily influenced by the licensing policy (13–15). Medical care is a life-and-death matter, which involves legal responsibility and ethical issues; thus, many countries have stipulated strict licensing policies toward it to facilitate supervision (16–18). The most critical one is limiting platform builders to hospitals. Despite the fact that there is a big market created by the strong needs of patients and more options for flexible practice for doctors, other market players longing to enter this field will find it quite difficult to obtain a license.

Licensing policy casts an enormous impact on the platform model. To be specific, the licensing policy has a great impact on the organizational form of the platform model, which is particularly prominent in telemedicine, an industry that is highly regulated (14, 19). At present, different licensing policies have generated different platform models in China, America, European countries, and other countries with the rapid development of telemedicine. Especially, in the 2000s, the Chinese government implemented a national electronic health (e-health) system *via* e-health records and other healthcare technologies (20). In addition, the government has developed supportive e-health policies, including the national e-health policy, the national multiculturalism policy for e-health, the national telemedicine policy, and the e-government policy. However, the stable and mature telemedicine platform models were far from being formed, mainly because these policies did not clearly stipulate which institutions can obtain telemedicine licenses. Until 2014, with the improvement of licensing policies, China's telemedicine platforms have ushered in vigorous development, and different platform models have gradually formed.

At present, the rapid growth of telemedicine platforms in China attracts research interest, but only a few studies summarize platform models and point out the relationship between licensing policy and platform models. Therefore, the research questions of this study are as follows: *(1) What telemedicine platform models exist in China? (2) How does the licensing policy affect the development of the platform models?* This study uses multiple case studies and the theory of policy implementation as mutual adaptation (ToPIAMA) to research the aforementioned questions.

The organizational structure of this study is as follows. Section 1 briefly introduces the relevant background of telemedicine and research questions. Section 2 reviews the previous related literature. Section 3 introduces the analytical methods and data sources. The next section summarizes the characteristics of the platform models combined with specific cases, analyzes the influence mechanism of licensing policy on the platform model, and carries out a validity test. Section 5 discusses the theoretical contribution of this research and puts forward policy recommendations to optimize the licensing policy and promote the development of the telemedicine industry. Finally, the limitations of this study and suggestions for future research are summarized.

## Literature review

### Telemedicine

Teleradiology and telepsychiatry are among the earliest telemedicine applications (21). Although the origin of telemedicine goes back a long way, the modern era of telemedicine began in 1968 when Massachusetts General Hospital in America began to provide remote clinical examinations for travelers and airport staff at Logan International Airport (22). High technology costs, poor image quality, lack of usage services, and the inability to integrate Internet medical care with mainstream healthcare services, most of which disappeared by 1980, led to a decade of hiatus in telemedicine activities (21, 22). It was not until the mid-1990s, due to the rapid growth of the Internet, that Internet-based medicine was once again seen as a relevant solution to the problems of licensing and quality of healthcare (23). Telemedicine has also attracted interest in the healthcare community due to its ability to reduce costs and save time for both patients and healthcare professionals (24, 25). In recent years, investments in start-up telemedicine service companies have skyrocketed. Thousands of hospitals are outsourcing selected gap services (e.g., nighttime and weekend coverage by teleradiology) and urgent services (e.g., telestroke services) (26).

In China, telemedicine began in the mid-1980s. The early Chinese telemedicine activities were mostly based on store-and-forward technologies, and real-time telemedicine was not used because the telecommunication infrastructure required was not available (27). Then, until the early twentifirst century, the early stage of telemedicine in China mainly focused on the demonstration of international cooperation among large medical institutions due to limited resources (28, 29). Recognizing the potential value of telemedicine, the Chinese government and medical institutions began to invest in telemedicine programs in the 2000s. Subsequently, relevant agencies established a few networks, such as the Golden Health Network, covering a number of cities and regions (29). Since the 2010s, with the rapid construction and popularization of information and communication technology in China, the development of telemedicine has also entered the fast lane, and many platforms and models have been born. Especially during the COVID-19 outbreak, telemedicine has played an important role in many regions in China, such as Sichuan and Shandong provinces (4, 30).

### Platform

Platforms are intermediaries between supplier and demander, which can provide licensing to goods and services around the world, lower transaction costs, expenditures for resources allocation, and support a multitude of new services (12, 31). Platforms are in many cases disrupting the existing organization of economic activity by resetting entry barriers, changing the logic of value creation and value capture, repackaging work, or repositioning power in the economic system (9, 32, 33). The emergence of the platform economy is reorganizing work, employment, and value creation (34) in which tools and frameworks based on the power of the Internet will frame and channel our economic and social lives (35).

The platform can be divided into different models according to different criteria, such as platform strategies (36), platform-mediated work (34), and the value creation mechanism (37). A platform model describes in an abstract way how platforms generate value for suppliers and demanders (38). Using models can master the challenges of developing digital platforms, which represent proven principles of already established platforms (39, 40). The different platform models can be used to analyze their sustainable value creation potential and business principles (41).

### Licensing and licensing policy

Licensing is essential for economic and social development and the formation of market structures (42). When social licensing is removed, it becomes clear to all that incurring both human and economic costs that sometimes can be irreparable (43). Occupational licensing poses substantial difficulties for job seekers and would-be entrepreneurs (44). Promoting simplification of licensing terms is an effective policy for fostering entrepreneurial activity and for making demanders affluent by increasing employment opportunities and by lowering prices (45). However, studies pointed out that more licenses need not result in a more competitive economic output (46).

### Summary

Platform-related research is maturing (47), and there is also literature on the typology of platforms in different industries. However, there are few studies on the platform models in the telemedicine industry. This study will analyze the platform models of telemedicine and study the influence mechanism of its key factor—the licensing policy—on platform models.

## Analytical methods and data sources

### Research methods

This study is a multi-case study. A case study is a methodology that explores a phenomenon or case in a natural setting, utilizing a series of methods to obtain in-depth data (48). Research questions such as “what” and “how” apply to case studies. The researcher argues that case studies focus on a single subject, and different methods of data collection could be used to obtain data (49). Thus, the case study approach was selected because it provides in-depth information about the telemedicine platform in China. Multiple cases are discrete experiments that serve as replications, contrasts, and extensions to the emerging theory (50). Based on the previous literature (51–53), the specific steps of the multi-case study method used in this study are as follows:

#### Confirming research questions

In the beginning, we wanted to learn about the influencing factors of the development of telemedicine. Based on the information obtained during the initial interviews, we found that licensing is a key factor, while the mechanism of its influence is poorly studied. Therefore, our research question is determined as: *(1) What telemedicine platform models exist in China? and (2) How does the licensing policy affect the development of the platform models?*

#### Case selection

By the first half of 2021, China has approved more than 1,600 telemedicine platforms (54). Although the total number of platforms is large, there are only three key types of platform providers due to the restrictions of licensing policies: medical institutions, Internet companies, and local government. Among them, the entry of Internet companies also needs to cooperate with medical institutions with high qualifications; otherwise, it is illegal. China does not have telemedicine platforms provided by insurance companies like other countries. Thus, this study selected three basic ones according to different platform providers to conduct multiple case studies, that is, Internet Hospital of the First Hospital of Jilin University, Good Doctor Online, and Zhejiang Provincial Internet Hospital Platform.

The criteria and reasons for selecting the aforementioned cases mainly are as follows. (1) The earliest explorers under the policy pilot. Local governments in China conduct policy pilots under the constraints of the central government. A group of platforms has been born in each pilot. (2) Survivors in the market economy. Most telemedicine platforms have been eliminated under the fierce market competition, and this research selects the survivors.

#### Comparative analysis

This article first introduces the selected cases, summarizes different platform models, and compares the platform models from analytical dimensions. The analytical dimensions are divided into primary dimensions and secondary dimensions (see Supplementary Table 1). The specific contents are listed in the table.

#### Validity test

This study uses telemedicine cases in America to test the summarized platform models and influence mechanisms. The reason for choosing America for the test is that both China and America have a large economy and population, have developed digital economic environments, and have strong demand for Internet diagnosis and treatment services. Compared with China, America has an earlier exploration history of telemedicine licensing policies and laws, and the relative system is more mature. The similarity of economic and social development effectively controls the irrelevant variables in the validity test, while the different development degree of licensing policy is the main variable in the test.

### Data sources

The two most important types of data are policy documents and case data. Policy documents are mainly sourced from Internet retrieval, including legal texts, domestic and foreign academic literature, dissertations, and newsletters. Documents relevant to China's telemedicine licensing policies have also been summarized in the study (see Supplementary Table 2).

Case data are sourced from both web retrieval and interview surveys. The details of the interview surveys are shown in Supplementary Table 3. Three interview surveys were carried out, the first two of which were one-on-one conversations with executives of a certain company. The first time was mainly to learn about the influencing factors of the development of the telemedicine industry. Then, the research questions were identified, and supplementary research was conducted. Under the introduction of this executive, the researchers held a symposium with executives of other two telemedicine companies for 2.5 h. Among these interviews, two of the interviewees were working or had worked in the company of two cases in this research. Through these interviews, the researcher collected relatively comprehensive data. The researchers used triangle verification to ensure the data validity, including using the websites or APPs of these telemedicine platforms, obtaining people's comments on these telemedicine platforms, and conducting multiple interviews.

During the research process, the researchers conducted a comparative analysis with other countries, mainly with America. Relevant information about telemedicine licensing in America mainly comes from network retrieval. The researcher searched relevant laws, regulations, and policy interpretation texts from the official websites of the U.S. Department of Health and Human Services and state health authorities. At the same time, academic literature in related databases such as PubMed and Elsevier was also searched.

### Theoretical framework

The ToPIAMA is the main theory applied in this study, which is used to analyze the influence mechanism of licensing policy on the platform model.

The theoretical model of policy implementation as mutual adaptation is a widely used and far-reaching theory in policy implementation theories, which was put forward by Mclaughlin (55). Its main content and implementation process are shown in Supplementary Figure 1. The core point is that the policy implementation process is essentially an interactive process between the policy implementer and those affected by the policy to adjust goals or means. The effectiveness of policy implementation fundamentally depends on the degree of behavioral adaptation between the policy implementer and those affected by the policy. Therefore, in the adaptation model of policy implementation, there are two parties that interact: one is the policy implementer and the other is those affected by the policy implementation. The process of policy implementation is the process of finding an adjustment strategy acceptable to both parties, which is also a process of continuous policy optimization.

The dynamic view of policy implementation in this theory is consistent with the characteristics that China's telemedicine licensing policy is developed and perfected according to national conditions. At the same time, the application of different adaptation models of policy implementation in this study is the development process of the platform model. In other words, the dynamic optimization of licensing policy promotes the continuous development of the platform model. Based on the above preliminary analysis, the interaction theory model selected by this study is relatively reasonable and highly feasible in analyzing the influence mechanism of licensing policy on the platform model.

### Case overview

Based on the current development status of telemedicine platforms in China, this study selected corresponding cases from the perspective of platform providers: medical institutions as platform provider (MIaPP), Internet companies as platform provider (ICaPP), and local government as platform provider (LCaPP).

As for MIaPP, the case selected is the Internet Hospital of the First Hospital of Jilin University (FHoJU). On 26 May 2020, FHoJU was approved to obtain the first telemedicine license in Jilin province and get the qualification to operate a telemedicine platform. Patients can have an interrogation diagnosis through the “Jiyitong” APP or the WeChat official account of FHoJU (see Supplementary Figure 2) and have follow-up consultation by sending online pictures and texts or by video interaction. By May 2022, the telemedicine of FHoJU has accumulated 4.21 million registered users, 1.05 million family account users, 9.5 million electronic bills, and more than 3 million outpatient appointments (56). On 31 May 2022, FHoJU was awarded the “Digital Health Demonstration Case of National Health Commission”.

As for ICaPP, the case selected is Good Doctor Online. Founded in 2006, Good Doctor Online has become one of the leading telemedicine platforms in China. Its main business includes graphic consultation, telephone consultation, remote expert outpatient service, transfer treatment appointment, post-diagnosis disease management, online follow-up consultation, outpatient information inquiry, and family doctor. Users can easily contact doctors through the Good Doctor Online application, PC version website (see Supplementary Figure 3), mobile version website, WeChat official account, WeChat applet, and other platforms and enjoy one-stop solutions to various medical issues such as online services and offline medical treatment. By July 2021, Good Doctor Online has served more than 72 million patients in total (57). Meanwhile, over 240,000 doctors have registered on the platform, and among these active doctors, 73% of them are from 3A hospitals (China's hospital classification indicates that these are the highest-level hospitals) (57, 58).

As for LCaPP, the case selected is the Zhejiang Provincial Telemedicine Platform. This platform is embedded with the electronic registration management system for medical institutions, doctors, and nurses. Officially released on 22 January 2019, it has become China's first platform integrating “service plus supervision”, as well as China's first platform to fully adopt electronic certificates. The Health Commission of Zhejiang province established this platform, at the same time, required telemedicine institutions within Zhejiang province to speed up the data interface with it (59). This platform has functions such as online consultation, chronic disease follow-up, prescription issuance, and drug distribution. It also provides services such as home care and pharmaceutical consultation. In the initial stage of the platform, it was set up on Alipay (see Supplementary Figure 4). Currently, apart from Alipay, it can also be accessed *via* WeChat mini-application and “Zheliban” application (a government affairs application developed by the Zhejiang Provincial Government). By March 2021, the number of registered users on this platform has reached 1.28 million, number of services 18.57 million; it has interfaced with 768 medical institutions and had 60,000 registered medical personnel, having unified the telemedicine service entrance in Zhejiang province (60).

## Results

Due to the strict supervision of medical care, the early development of telemedicine in China was almost illegal. Document No. 02 in 2014 allowed medical institutions to provide diagnosis and treatment services to non-admitted patients for the first time. Document No. 05 in 2018 made regulations on the licensing of Internet diagnosis, treatment activities, and telemedicine platforms. These documents had effectively promoted the development of telemedicine in China. Subsequent relevant policies have either encouraged the development or strengthened the supervision, providing entry opportunities for telemedicine platforms. Telemedicine activities recognized by policymakers evolved from online drug sales to Internet hospitals, remote diagnosis, and treatment and then to derived services such as Internet nursing and family doctors.

### Characteristics of telemedicine platform models in China

Model is a certain realization path and form of knowledge in the process of solving problems (40, 51). Platform model is the organizational structure and accessible method that rely on the organizational form of the platform to solve specific conversion needs of the industry and improve the efficiency of resource allocation (61, 62). To clear up China's telemedicine platform model is of great significance for both deepening the understanding of the platform and guiding its development in the future.

#### Model of MIaPP

Under the model of MIaPP, a single medical institution builds its own telemedicine platform (see Supplementary Table 4) using the institution's own medical resources and only conducting consultation online, which essentially is the extension of the physical medical institution. The “medical institution” here refers to hospitals, primary medical and health institutions, professional public health institutions, and other institutions that have medical and health resources [according to the *Implementation Rules for the Regulations on the Management of Medical Institutions* (63)]. To facilitate supervision, all online records are stored on a telemedicine supervision platform. Local governments without such a supervision platform cannot grant a license to telemedicine (see Document No. 04). Famous MIaPPs include the telemedicine of FHoJU, the Internet Hospital of the China-Japan Friendship Hospital, and the Internet Hospital of the First Affiliated Hospital of Sun Yat-sen University.

The platform itself has the following characteristics. The platform provider is a single regional medical institution, different from some telemedicine platforms provided by medical groups in America. In 2015, the Chinese government issued a series of policies (Documents 03, 04, etc.) and allowed medical institutions to establish telemedicine platforms. Since then, relying on a large number of medical institutions in China, this model has been developing rapidly, and the platform functions keep being optimized. At present, remote diagnosis and treatment with focuses on the follow-up of common diseases and chronic diseases and other auxiliary functions have been realized. Since all platforms are isolated from each other in this model, patient data cannot be shared between different platforms. However, it also suggests that large-scale data breaches are almost impossible. Under this model, the diagnosis and treatment services as well as the drugs are charged in accordance with government-guided prices and hospital charging standards, which indicates that the bargaining power of the platform is weak. The reputation mechanism of the platforms is mainly developed by word of mouth; for example, people can get comments about the platform from their friends. Overall, this model has a far-reaching influence in China.

Suppliers and demanders have the following characteristics. Doctors, as the suppliers, come from medical institutions. According to the research data from the China Medicine website (http://med.china.com.cn/), a national news site, 69.5% of the suppliers come from public hospitals, while only 30.5% are from private hospitals and other institutions. Public hospitals are at the disposal of core medical service elements such as doctors, equipment, and venues and also have strong offline service capabilities; thus, they are the backbones in the providing of online services. The advantages of private hospitals and other institutions lie in the differentiated and high-quality services they can provide through Internet hospitals and that they can activate the supply side through the reasonable distribution of benefits, creating a personal brand for platform star specialists or multi-site GPs. Currently, this model has been widely adopted by local governments in China, and the user number has reached as high as 620 million. The cost of medical treatment is controlled by the government and is relatively low. In addition, public hospitals have incorporated Internet diagnosis and treatment into the scope of medical insurance, which can further reduce the costs borne by patients.

The sustainability of this model has been questioned. Internet diagnosis and treatment activities are provided by a single medical institution, as a result, medical resources are isolated from each other, and it is difficult for patients to make referrals. The localization nature of medical insurance has also weakened its capability to break through geographical space limitations (23). However, it has been deeply rooted in China's telemedicine industry and has the largest number of business outlets and the largest application scale, helping to ease patients' difficulty in getting medical services to a large extent and achieving strong usage intention by patients. Under this model, most of the doctors are from public hospitals, and there are clear charging standards for diagnosis and treatment; in addition, the licensing policy that only allows doctors to conduct Internet diagnosis and treatment in their own medical institutions limits the income of doctors, especially some specialists, and has weakened their usage intention. In addition, the self-built Internet hospital by a single medical institution lacks economic efficiency for the limited overall number of suppliers and demanders. From the perspective of the platform economy, the cost of operating a telemedicine platform with 1,000 doctors and one with 10,000 doctors is almost the same (11, 12). From the perspective of the supplier, the higher the cost of the platform, the lower the profit; correspondingly, doctors' commission is not high, and thus, doctors are not motivated to use the platform. The shrinking of the suppliers leads to the loss of the demanders and further affects the sustainable development of the platform. Overall, the sustainability of this platform model is comparatively weak.

#### Model of ICaPP

Under the model of ICaPP, Internet companies establish the platform by relying on and connecting with physical medical institutions (see Supplementary Table 4). Compared with the former one, this model better integrates medical and health service resources. In addition to Good Doctor Online, there are also a number of successful Chinese telemedicine companies, such as Ping an Good Doctor, Lilac Doctor, and 春雨医生.

The platform itself has the following characteristics. The platform provider is the Internet company. This model emerged the background when Chinese Internet companies developed rapidly and the policy background that incensing policies (**Documents No. 03, 04, etc**.) gradually allowed Internet companies to establish a medical platform and carry out prescribed medical activities. Since 2015, a large amount of capital has poured into China's telemedicine industry, and people's habit of using online medical treatment is gradually developed. Under the leadership of Internet companies and *via* the market-oriented operation means, in addition to the diversification of the main remote diagnosis and treatment activities, the platform has also developed health tracking, management, service, timely intervention, and other health management value-added functions, gradually forming a telemedicine enterprise service ecosystem (64). However, Internet companies may conduct illegal transactions on patient data driven by profit, which exacerbates the risk of privacy leakage. Under this model, except that the prices of medicines are regulated by the government, doctors' outpatient fees are relatively flexible, which shows that the platform has relatively strong bargaining power. In addition, the platform enables patients to learn about the doctor's reputation through the online evaluation system, and patients can express their gratitude to the doctor through online rewards.

Suppliers and demanders have the following characteristics. Internet companies provide platforms to integrate the medical resources of physical hospitals, so most suppliers are part-time doctors. At present, most Chinese telemedicine companies have opened registration and services to all regions in China. With the user scale reaching 298 million and the growth rate still keeping growing, this model has great potential for development. Under Internet companies' management, the charging for diagnosis services has a certain degree of autonomy and flexibility. That is, the diagnosis and treatment service cost will be dynamically adjusted according to the supply and demand relationship between doctors and patients; thus, the charge is relatively high. In addition, doctors can choose the working platform and working time more flexibly.

This model is more sustainable. In this model, Internet diagnosis and treatment activities can be carried out more openly and across regions (in the model of MIaPP, Internet diagnosis and treatment activities are generally limited to a certain province), and it is relatively easy to refer patients as well as to establish a neutral word-of-mouth system. All these help to enhance the patient's willingness to use the platform to a greater extent and attract a large number of new patients. In the guidance and distribution of patients, Internet companies and medical institutions can form upstream and downstream industry chain relationships. Through telemedicine companies, medical institutions can expand their channels to attract patients, which in return, simulates their willingness to use the platform more. At the same time, the charging for the doctor's diagnosis and treatment services is relatively flexible, e.g., “star specialists” charge higher. The incentive mechanism will naturally make doctors have a higher willingness to use the platform. This platform model has good development prospects and strong sustainability.

#### Model of LGaPP

Under the model of LGaPP, local governments assign medical institutions to build a regional telemedicine platform (see Supplementary Table 4). Compared with the first model, this model can integrate regional medical resources; in other words, this model is a further development and extension of the first model. In China, in addition to the Zhejiang Provincial Telemedicine Platform, other local governments also provide telemedicine platforms, including Sanming Municipal Government and Tianjin Municipal Government.

The platform has the following characteristics. It develops according to the encouragement and guidance of the licensing policy in China (Documents No. 06, 07, etc.). After the release of Document No. 06, local governments in China began to actively explore this field. Since 2019, this model, with functions such as online follow-up diagnosis and online consultation, began to prosper. On 8 October 2021, the release of Document No. 09 marked the successful pilot and official promotion of this model in China. Since local governments have the attributes of the public sector, patient data cannot be illegally traded, but there is a possibility that patient privacy may be leaked because of technical problems and improper operations. Similar to the model of MIaPP, the bargaining power of the platforms in this model is weak. Local governments develop doctors' reputation mechanisms through promotion mechanisms and evaluation systems.

Suppliers and demanders have the following characteristics. Doctors, the suppliers, come from participating hospitals in the region and are qualified to provide Internet diagnosis and treatment services. Patients, the demanders, have exceeded 25 million in number, mainly distributed in the aforementioned pilot provinces and cities. These characteristics have not only proven the rapid development of this model but also reflected that it has only been established and implemented in a small area in China. If the platform is under the management of the government, the charging for diagnosis and treatment services is strictly subjected to supervision and guidance. The costs are relatively low and can be fully included in medical insurance.

The sustainability of this model needs to be further examined. The model is currently in the stage of a successful pilot and is to be promoted nationwide, but its reproducibility has yet to be tested in practice. However, what is certain is that this model is convenient to refer patients, helping to integrate doctor resources and patient groups in the region, and is able to improve doctors' and patients' willingness to use the platform. However, two points still remain to be changed: the limited income of doctors from the diagnosis and treatment services and the fixed-point practice. Thus, doctors' willingness to use this platform model is still weak.

### Analysis of the influence mechanism of licensing policy on platform model

Licensing policy is the premise and key to the development of a telemedicine platform, which can systematically promote the formation of its ecology (3–5). The process of implementing platform licensing policy is the process of exerting its influence. Therefore, it is necessary to start with the policy implementation process. The implementation process of licensing policy involves two objects: policy implementer and the affected people. For the telemedicine licensing policy, the policy implementer is the platform provider, and the affected people are mainly the suppliers and the demanders. At present, the stipulation of China's licensing policy toward these two objects is as follows.

For policy implementers (i.e., platform providers): Documents No. 02, 03, and 06, respectively, permit medical institutions, Internet companies, and local governments to provide telemedicine platforms.

For affected people—the suppliers (i.e., doctors): Document No. 05 explicitly stipulates that the admission requirements for doctors are “physicians who provide services in Internet hospitals shall ensure that they have completed the diagnosis and treatment work specified by the main practice institutions” and “physicians who carry out Internet diagnosis and treatment activities shall obtain corresponding practice qualifications in accordance with the law, having more than 3 years of independent clinical work experience, and having the consent of the medical institution where his or her practice is registered.” The positioning of telemedicine in China's medical industry is to do auxiliary service, so doctors must first complete offline diagnosis and treatment work prescribed by the practice institution.

For affected people—the demanders (i.e., patients): Document No. 05 explicitly stipulates: “Internet diagnosis and treatment activities shall not be carried out for initial-diagnosis patients”.

According to the ToPIAMA, the relationship of the main objects involved is shown in Supplementary Figures 1, 5. Specific to this study, the analysis of the above three environmental factors of licensing policy is as follows: (1) Political environment: The relevant policy system in China is imperfect, so it cannot meet the needs of the rapid development of telemedicine. The deficiency of top-level design in terms of information security, privacy protection, and online practice certification has hindered the development of telemedicine. Therefore, relevant policies are usually implemented in the form of administrative orders. (2) Economic environment: China's good economic environment provides high-quality external conditions for the development of telemedicine. A large sum of social capital has poured into the telemedicine market. Internet companies have been actively developing the telemedicine industry and constantly innovating operation models, which has injected vitality into the development of telemedicine. Therefore, the market mechanism is an important way to implement relevant policies. (3) Social environment: Currently, the aging problem in China is getting more and more severe; as a result, people's demand for continuous, regular, and normalized health management and family rehabilitation services keep increasing. Medical insurance has gradually become an important factor affecting the implementation of licensing policies. In the telemedicine industry, the constraints or incentives of licensing policy on platform, suppliers, and demanders are realized by the platform provider who formulates implementation rules, defines approval standards, and stimulates promotion. The implementer of licensing policy is the platform provider. Therefore, the influence of licensing policy on the platform model is manifested as the platform provider's influence on the platform model. Under this context, administrative orders, market-oriented mechanisms, and medical insurance are the main means for the platform provider to cast influence upon doctors and patients, and during this process, the policy implementer and the affected people adapt to each other, forming a relatively stable while continuously evolving platform model.

In this study, the influence mechanism of licensing policy on the telemedicine platform model mainly has the following two paths (see Supplementary Figure 5).

First, the licensing requirements of the policy implementer (i.e., platform provider) affect the platform model. Due to the guidance and support of the licensing policy, medical institutions, Internet companies, and local government have become the main force in the construction of China's telemedicine platform. However, it is undeniable that the establishment of the licensing policy system in the field of telemedicine in China has not yet followed the pace of industry development, and the lack of relevant legislation and institutional conflicts have hindered the development of the industry (65). For example, Chinese law currently does not authorize insurance companies to engage in healthcare-related activities [according to Article 95 of the *Insurance Law of the People's Republic of China (2015 Revision)* (66)]. Therefore, the model of insurance companies as a platform provider, which is an effective model in America, is hard to be carried out in China. The current, not up-to-date licensing policy has determined that there are only three types of telemedicine platform providers in China: medical institutions, Internet companies, and local governments. On a deeper level, providers are not only platform providers but also platform leaders who directly determine the establishment and formation of the telemedicine platform model.

Second, the policy implementer affects the platform models by restricting licensing to the suppliers and the demanders of the platform. The affected people will affect the formation and development of the platform model, which will be explained below from both the supplier and the demander aspects. As to suppliers, compared with offline diagnosis and treatment, online diagnosis and treatment have a higher threshold for doctors to enter, which, on the one hand, effectively ensures the quality and safety of Internet diagnosis and treatment services but, on the other hand, greatly restricts the entry of platform suppliers. At the same time, doctors' Internet diagnosis and treatment service are fixed-point and single-point because medical institutions will restrict doctors to provide telemedicine services within their own institutions. As a result, the flow of platform medical resources and the maturity of the platform model will be restricted. As to demanders, the prohibition of patients' initial diagnosis is for the sake of safety, but the “one-size-fits-all” approach regardless of disease type and the specific situation will lead to the loss of patients with common diseases, chronic diseases, and so on, and patients that are far from the medical institution in location and those who have tight time. This limits the usage of platform demanders. Therefore, the licensing policy casts influence upon the affected people through the policy implementer, which in turn affects the formation and development of the platform model. In other words, the licensing policy and its implementation indirectly affect the sustainable development of the platform model through the affected people.

Based on the aforementioned analysis, this study summarizes two paths of China's telemedicine industry licensing policy to influence the platform model and draws the conclusion that licensing policy can not only stipulate platform licensing requirements directly determine the establishment of the platform model but also indirectly affect the sustainable development of the platform model through the supplier and the demander of the platform.

### Validity test

Due to the network effect and scale economy effect of the Internet, it is impossible for a country with a small population or a backward information infrastructure to incubate a large telemedicine platform. Taking the development of the telemedicine platform in America as an example can effectively carry out the validity test.

Telemedicine platform, as the main carrier for remote diagnosis and treatment activities, is mainly established by Internet companies, hospitals (or medical groups), or third parties such as insurance companies in America (67). The platform is built by both one party alone and by multiple parties in cooperation. America has an earlier history of telemedicine development, and its laws and regulations are more complete and mature. At present, laws involved with telemedicine in America mainly include *Federal Fraud-and-Abuse Laws, Anti-Kickback Statute*, and the *Stark Law*. (65). Currently, the telemedicine market in America is highly concentrated with Internet companies outshining others. Amwell, MDLIVE, and Teladoc occupy most of the market share. Amwell, in particular, possesses 150 million medical insurance users, serves more than 55 medical insurance plans, has 240 medical groups, and over 2,000 hospitals in total. American insurance companies and hospitals (or medical groups) have also set up telemedicine platforms, but most of these platforms are technically supported by the aforementioned three Internet companies. Few insurance companies will establish their own independent telemedicine platform, for instance, Anthem Insurance (Anthem) has established its own telemedicine platform “LiveHealth Online” through an independent subsidiary.

In terms of platform model classification, America and China are different. According to the induction method of this study, telemedicine platforms in America can be classified into the model of Internet companies as platform provider, the model of medical group as platform provider, and the model of insurance company as platform provider. The “model of medical group as platform provider” is similar to China's model of LGaPP. It can be seen that, in America's relatively mature telemedicine system, there is no model of MIaPP, indicating that this model is less sustainable and has been eliminated during the evolution of the platform model. On the contrary, the model of ICaPP has become main stream in America's telemedicine industry, and “model of medical group as platform provider” also exists widely in America, indicating that these two platform models are highly sustainable. In addition, American law allows insurance companies to enter the telemedicine industry, incubating a new feasible model of the medical group as a platform provider. The development of the telemedicine platform model in America shows that the inductive analysis of the platform model in this study is correct and feasible.

In terms of licensing policies affecting platform models, America and China are the same. Currently, multiple telemedicine platform providers coexist in America as a result of the laws and regulations related to telemedicine. These multiple providers thus determine the telemedicine platform model in America. Furthermore, the permit for initial diagnosis service in telemedicine has been applied for many years in America. On 27 May 2017, Texas Governor passed the state's telehealth legislation bills (i.e., Senate Bill SB1107 and House Bill HB2697), repealing the regulation that doctors can only provide telehealth services to patients after face-to-face contact; since then, all states in America have been supportive for the permit of Internet initial diagnosis. By November 2019, 49 state legislatures and medical boards in the District of Columbia, Puerto Rico, and the Virgin Islands had required doctors who practice telemedicine to be licensed in the state where the patient is located; 12 state legislatures had issued telemedicine licenses that allow in-state and cross-state telemedicine practice; six states had required that physicians doing cross-state practice should register. The above regulations effectively promote doctors' and patients' willingness to use the platform and are conducive to the sustainable development of the telemedicine platform.

In summary, while the models are different, the influence mechanism is applicable. The aforementioned analysis shows that the influence mechanism of licensing policies (regulations) on the platform model in this study is effective.

The validity test has verified platform models and the influence mechanism summarized by this study, indicating that the results of this study are valid and reliable.

## Discussion

### Theoretical contribution

#### Supplement the research on telemedicine platforms

Most of the previous studies on telemedicine were carried out from the perspective of its development process (22, 68), its characteristics (26, 69), and economic and social benefits (25, 33), and there were few studies summarizing and professionally analyzing telemedicine from the perspective of platform formation and development. This article systematically studies the characteristics of telemedicine platform models and discusses the economic effect of telemedicine platforms, thus effectively supplementing the related research on telemedicine platforms.

#### Enrich platform research from the perspective of the platform model

Although the research on the platform model has formed a system, it is carried out from the following aspects: internal platforms (within the firm), supply chain platforms (within a supply chain), industry platforms (industry ecosystems), and multi-sided markets or platforms (industries) (36, 70). When studying the industry platforms, most of the research is from the perspective of the platform formation process (36, 71) and operation mechanism (72–74). There are few studies that divide the industry platform into platform models. The research on the platform model in this study fills this gap, and it has enlightenment and reference significance for subsequent researchers to conduct research on an industry platform.

#### Expand the research scope of the licensing policy

Previous studies on licensing policy were mostly limited to the scope of the market (75, 76), company (77, 78), and industry (42, 79). There is little literature on the mechanism of platform licensing. This article studies the content and characteristics of telemedicine licensing policy and deeply examines its impact on the development of the platform models.

### Practical inspiration

#### Streamline and integrate the hospital's self-built Internet platform

The platform has a scale effect and a network effect, and it is difficult for a telemedicine platform built by a single medical institution to exploit the advantages to the full. Streamlining and integrating these separate platforms can improve the operational efficiency of platforms and facilitate the formation of a platform ecosystem. The model of MIaPP is a transitional model in the development of a telemedicine platform and a product of an underdeveloped platform organization. The formation mechanism and the inherent characteristics of the platform (32) determine that this model will eventually evolve and streamlining and integrating the platform is one of the evolution paths.

#### Dynamically optimize telemedicine licensing policy

According to the ToPIAMA, the process of policy implementation is the process of finding an adaptation strategy acceptable to both the policy implementer and the affected people, which is also the process of optimizing licensing policy; thus, the platform model is the product of adaptation by both parties. The formulation and implementation of telemedicine licensing policies are a dynamic process that cannot be accomplished overnight. The government should continuously improve the elements in the licensing policy that hinder the sustainable development of the platform based on the feedback from doctors and patients and, at the same time, explore and develop the platform model based on national conditions and international advanced experiences. For example, the initial diagnosis of some special diseases is conducted, the scope of diagnosis and treatment was moderately expanded (24), patient information is effectively protected, the ownership of electronic medical record information is clarified, and the legal relationship and responsibility distribution of participants in the telemedicine field is stipulated.

## Conclusion

Through the aforementioned research, this study draws the conclusion that different licensing policies will affect the formation of different platform models. The licensing policy of the telemedicine platform not only directly determines the establishment and formation of the platform model by the platform provider but also indirectly affects the sustainable development of the platform models through the platform provider's influence on the suppliers and the demanders. At the same time, this study has the following limitations. First, the telemedicine model has not been further analyzed quantitatively, for example, the platform operation efficiency, the proportion of doctors with different professional titles, and the distribution of patients in different platform models have not been analyzed with relevant data to further explore the platform model. Second, due to the number of samples selected is limited, there may be a potential problem of sample bias. Despite the aforementioned limitations, this study provides the theoretical basis and practical enlightenment for developing countries to build telemedicine platforms and provides theoretical support for the development of a platform economy in the medical industry from the perspective of licensing policy.

## Data availability statement

The original contributions presented in the study are included in the article/Supplementary material, further inquiries can be directed to the corresponding author.

## Ethics statement

Ethical review and approval was not required for the study on human participants in accordance with the local legislation and institutional requirements. Written informed consent from the participants was not required to participate in this study in accordance with the national legislation and the institutional requirements.

## Author contributions

ZW: investigation, data collection, methodology, writing—review and editing, and funding acquisition. RX: data collection, methodology, and writing—original draft. YLiu: data curation and formal analysis. YLi: data collection and formal analysis. All authors contributed to the article and approved the submitted version.
