# Supplementary material for: Licensing policy and platform models of telemedicine: A multi-case study from China
Source: Front Public Health. 2023 Feb 2;11:1108621. doi: 10.3389/fpubh.2023.1108621 (PMC9932510; doi:10.3389/fpubh.2023.1108621)
Supplement: Supplementary file 1 [file Data_Sheet_1.docx]

Supplementary Material

Licensing Policy and Platform Models of Telemedicine:

A Multi-Case Study from China

Zhong Wang, Rui Xu*, Yan Liu, Yiming Li

*** Correspondence:** Rui Xu: xur2022@163.com

# Supplementary Data

None.

# Supplementary Figures and Tables

## Supplementary Figures

**Figure 1: Theoretical Model of Policy Implementation as Mutual Adaptation**


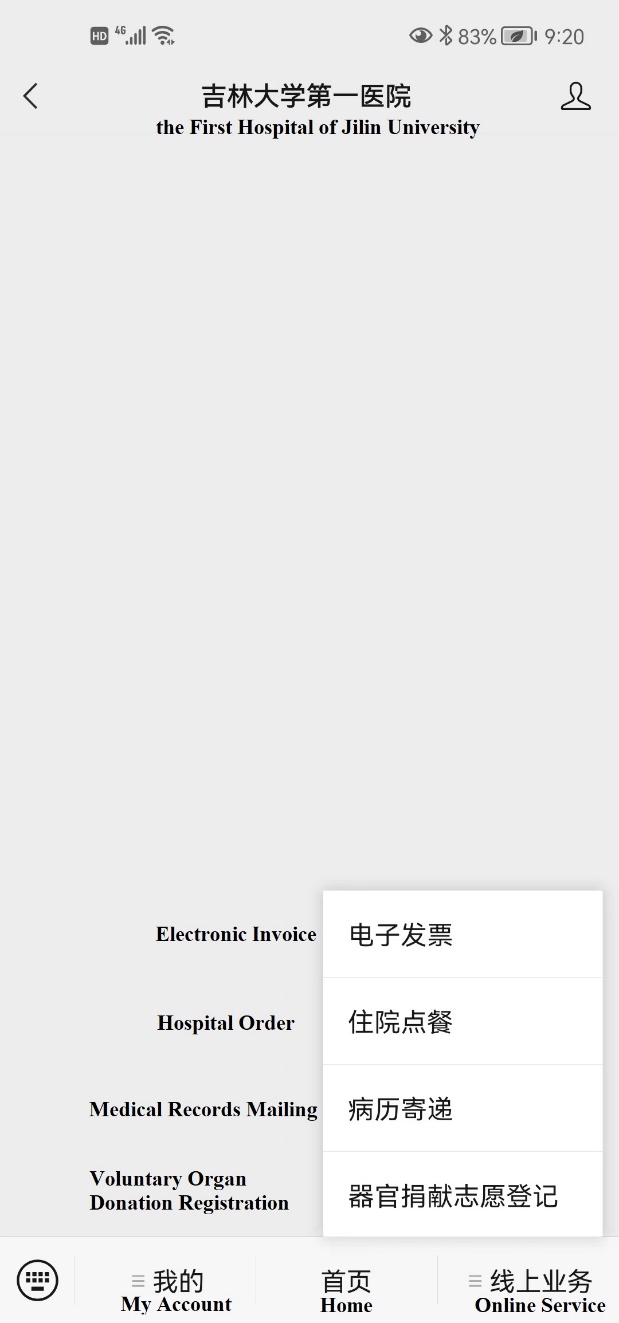

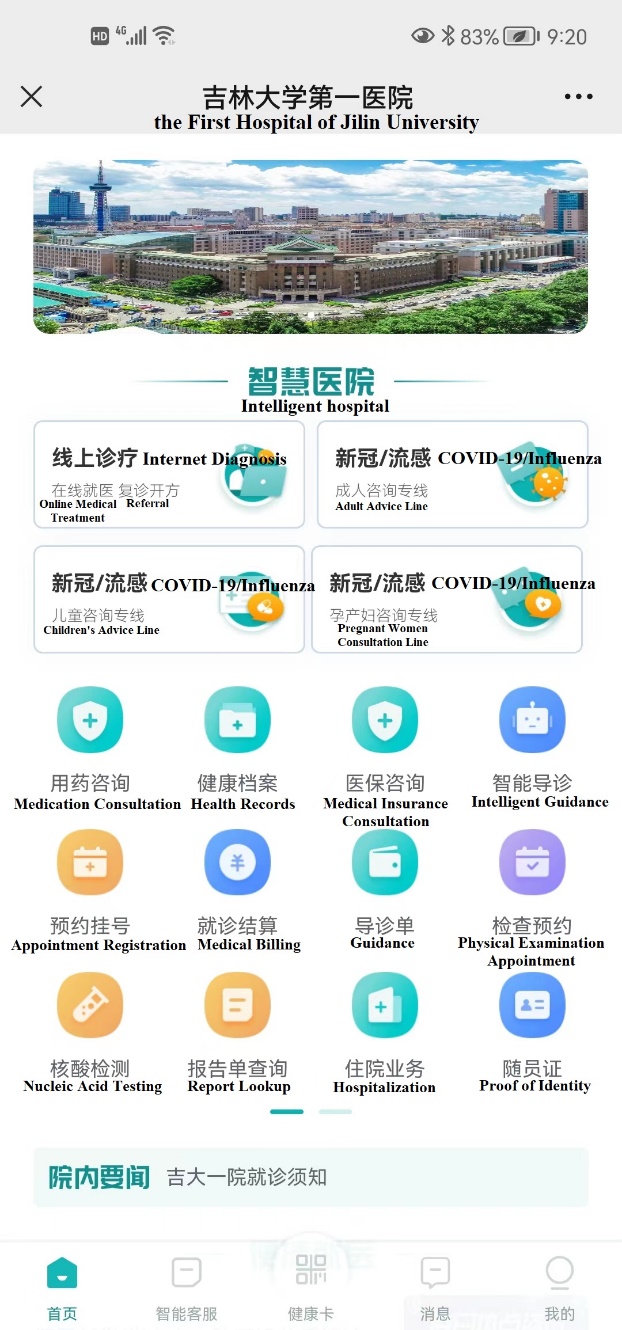


**Figure 2: Interface screenshot of the Wechat official account of the Telemedicine of the First Hospital of Jilin University**

Note:

1. Left is the main interface of the Wechat official account, right is the interface of the Home.
2. Access time: June 19, 2022.


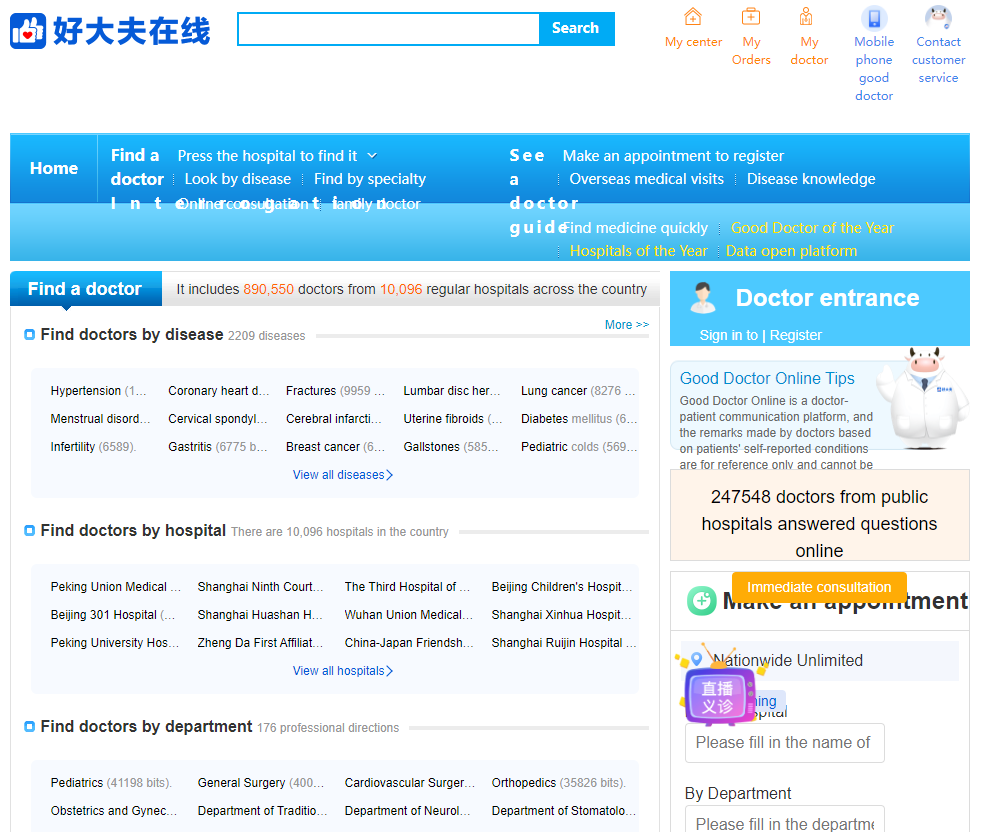


**Figure 3：Screenshot of PC version website of Good Doctor Online**

Note: Access Time: June 19, 2022


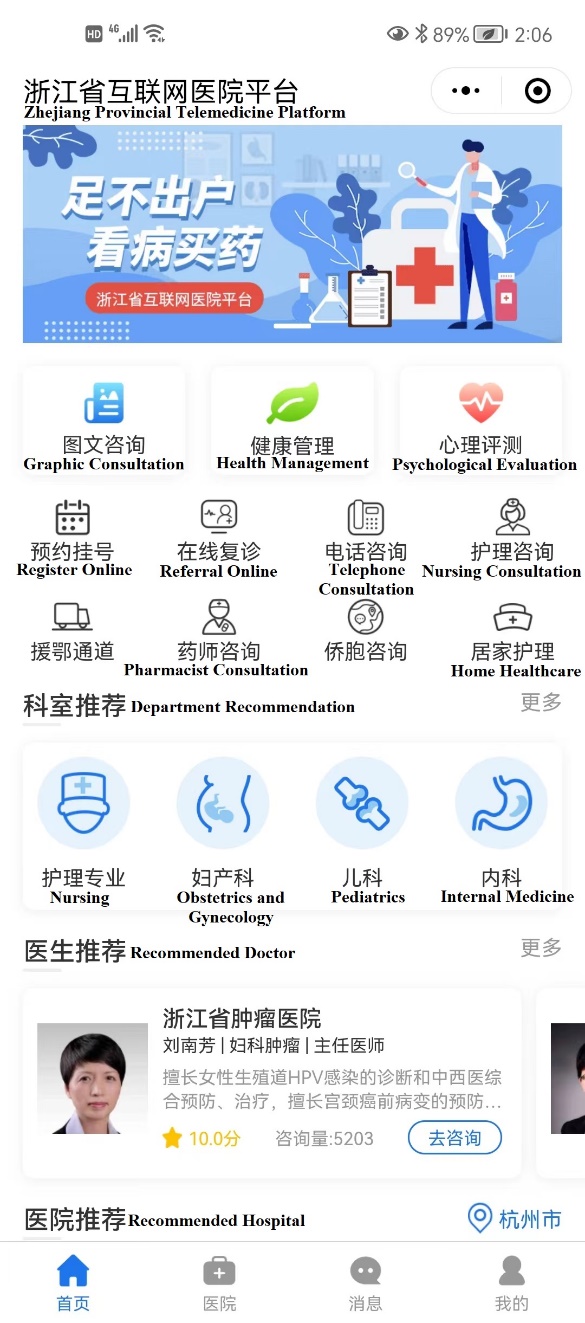


**Figure 4: Screenshot of patient’s entry on Zhejiang Provincial Telemedicine Platform in Alipay**

Note:

Access Time: June 7, 2022

**Figure 5：Influence Mechanism of Licensing Policy on Platform Model**

## Supplementary Tables

**Table 1: Analytical dimensions of platform model**

| **Primary dimension** | **Secondary dimension** |
| --- | --- |
| Platform itself | Rise time |
|  | Function realization |
|  | Privacy protection |
|  | Bargaining power |
|  | Reputation mechanism |
| Supplier | Doctor sources |
|  | Resource specificity |
| Demander | User scale |
|  | Application regions |
|  | Diagnosis and treatment costs |
| Sustainability of  Platform model | Usage intention of supplier |
|  | Usage intention of demander |
|  | Sustainability of platform model |

**Table 2: Documents relevant to China’s telemedicine licensing policy**

| **Docu-ment**  **No.** | **Document Name** | **Implementation Time** | **Formulation Department** | **Licensing Policies** |
| --- | --- | --- | --- | --- |
| 01 | *Notice on Strengthening the Management of Telemedicine Consultation* | 1999-1-4  (2020-12-30 invalid) | Former Ministry of Health of the P.R.C. | 1. Clarify that telemedicine consultation is still in start-up stage in China  2. Stipulate that **medical institutions** must obtain a license to conduct telemedicine consultation  3. Stipulate that **Physicians** must obtain the title of associate professor or above to conduct telemedicine consultation |
| 02 | *Opinions on Promoting Telemedicine Services in Medical Institutions* | 2014-8-21 | Former Health and Family Planning Commission of the P.R.C. | The platform established by **medical institutions** permitted to provide remote diagnosis and treatment services to non-admitted patients for the first time |
| 03 | *Guiding Opinions on Actively Promoting “Internet Plus” Actions* | 2015-7-4 | State Council | 1. Propose to develop telemedicine health services  2. Support the **third-party organization** to build medical information sharing service platform for the first time  3. Encourage **Internet companies** to cooperate with **medical institutions** to establish medical network information platform |
| 04 | *Opinions of the General Office of the State Council on Promoting the Development of “Internet Plus” Medical Health* | 2018-4-28 | Office of the State Council | 1. Allow the development of Internet hospitals to rely on medical institutions  2. Support the qualified **third-party organization** to carry out telemedicine, health consultation, and health management services |
| 05 | *Administrative Measures for Telemedicine (Trial Implementation), Administrative Measures for Internet Hospitals (Trial Implementation), Regulations for the Administration of Telemedicine Services (Trial Implementation)* | 2018-7-17 | National Health Commission of the P.R.C., National Administration of Traditional Chinese Medicine | 1. Explicitly stipulate the entry requirements for telemedicine **physicians**  2. Explicitly stipulate the entry requirements for telemedicine **patients** |
| 06 | *Notice on Priorities in the Construction of the Hierarchical Diagnosis and Treatment System* | 2018-8-7 | National Health Commission of the P.R.C., National Administration of Traditional Chinese Medicine | 1. Promote **all medical associations** to develop telemedicine services with focus on the remote and poverty-stricken areas; and clearly stipulate the time requirements  2. Encourage medical associations and communities to realize the initial- diagnosis, remote consultation, and two-way referral at the grassroots level |
| 07 | *Notice on Printing and Distributing Administrative Measures for Medical Associations(Trial)* | 2020-7-9 | National Health Commission of the P.R.C., National Administration of Traditional Chinese Medicine | Clarify the concept of “medical association” and encourage its gradual promotion of Internet diagnosis and treatment |
| 08 | *Opinions on Promoting the High-quality Development of Public Hospitals* | 2021-6-4 | Office of the State Council | 1. Emphasize that public hospitals are the backbone of China’s medical service system  2. Propose that public hospitals should focus on the development of telemedicine and Internet diagnosis and treatment |
| 09 | *Implementation Opinions on Widely Promoting the Experience of Sanming City, Fujian Province and Deepening the Reform of the Medical and Health System* | 2021-10-8 | Leading Group of State Council for Deepening the Reform of Medical and Health System | The successful experience of promoting the “local government as platform provider” marks the successful pilot and official promotion of this model in China |
| 10 | *Notice on Printing and Distributing the Detailed Rules for the Supervision of Telemedicine (Trial)* | 2022-3-15 | General Office of National Health Commission of the P.R.C, Office of the National Administration of Traditional Chinese Medicine | On the basis of **Document No. 04**  1. Clarify the detailed requirements for patients’ access to Internet diagnosis and treatment  2. Clearly stipulate to assess the medical staff of Internet diagnosis and treatment, and establish a licensing and dismissing mechanism |
| 11 | *Notice on Notification and Commendation of Typical Digital Health Cases (Second Batch)* | 2022-5-31 | General Office of National Health Commission of the P.R.C | The First Hospital of Jilin University ranks as one of the 10 demonstration cases of “Internet+medical and health” development that can be replicated and promoted, marking the successful practice of the “model of medical institutions as platform provider” |

Note (licensing time: 18 June, 2022):

The relevant information in the document here is translated from the Chinese government website. In order to avoid unnecessary misunderstanding, the information sources and the original names of the document are annotated as follows。

01: https://www.zryhyy.com.cn/ycyl/c103801/201706/f005deb50442448b9820f7fb3a31ba3c.shtml

《关于加强远程医疗会诊管理的通知》

02: http://www.gov.cn/gongbao/content/2014/content_2792664.htm 《关于推进医疗机构远程医疗服务的意见》

03: http://www.gov.cn/zhengce/content/2015-07/04/content_10002.htm 《关于积极推进“互联网+”行动的指导意见》

04: http://www.gov.cn/zhengce/content/2018-04/28/content_5286645.htm 《国务院办公厅关于促进“互联网+医疗健康”发展的意见》

05: http://www.gov.cn/zhengce/zhengceku/2018-12/31/content_5435436.htm 《互联网诊疗管理办法（试行）》《互联网医院管理办法（试行）》《远程医疗服务管理规范（试行）》

06: http://www.gov.cn/zhengce/zhengceku/2018-12/31/content_5435248.htm 《关于进一步做好分级诊疗制度建设有关重点工作的通知》

07: http://www.gov.cn/zhengce/zhengceku/2020-07/18/content_5528009.htm 《关于印发医疗联合体管理办法（试行）的通知》

08: http://www.gov.cn/zhengce/content/2021-06/04/content_5615473.htm 《关于推动公立医院高质量发展的意见》

09: http://www.gov.cn/zhengce/zhengceku/2021-10/15/content_5642920.htm 《关于深入推广福建省三明市经验 深化医药卫生体制改革的实施意见》

10: http://www.nhc.gov.cn/yzygj/s3594q/202203/fa87807fa6e1411e9afeb82a4211f287.shtml 《关于印发互联网诊疗监管细则（试行）的通知》

11: http://www.nhc.gov.cn/guihuaxxs/gongwen1/202205/7879709a521048a7bdbce1a9bee9729b.-shtml 《关于通报表扬数字健康典型案例（第二批）的通知》

**Table 3: Details of Interview Surveys**

|  | Interviewee | Form | Duration | Date | Main Content |
| --- | --- | --- | --- | --- | --- |
| First Interview | A | One-to-one telephone interview | 1 hour | Afternoon of April 23, 2020 | Basic situation of Good Doctor Online; the development process, influencing factors and main models of telemedicine |
| Second Interview | A | One-to-one face-to-face interview | 1 hour | Afternoon of June 18, 2020 | The impact of the licensing policy on the telemedicine platform, the suppliers and the demanders |
| Third Interview | A, B, C | Symposium | 2.5 hour | Afternoon of August 6, 2020 | The basic situation of the respondent’s company, their views on the telemedicine industry; the impact of the licensing mechanism on the company and the industry |

**Table 4: Profile of Telemedicine Platform Models in China**

| **Model name** | | **Model of MIaPP** | **Model of ICaPP** | **Model of LGaPP** |
| --- | --- | --- | --- | --- |
| **Platform**  **itself** | Provider | Single medical institution | Internet company | Local government |
|  | Rise time | 2015 | 2015 | 2019 |
|  | Function realization | Remote diagnosis and treatment (e.i., occasional diagnosis and follow-up diagnosis of common diseases and chronic diseases)，health consultation，health management | Online registration, remote interrogation (e.i., graphic interrogation, telephone interrogation, video interrogation), health management (health tracking, management, service and timely intervention) | Online consultation, online follow-up diagnosis, other consultation services (e.i., online guide diagnosis, check-up appointment, smart payment, etc.)* |
|  | Quantity rate | 70%** | 20%** | 10%** |
|  | Bargaining power | Weak | Strong | Weak |
|  | Reputation mechanism | By word of mouth | By online evaluation system | By the government's promotion mechanism and evaluation system |
| **Suppliers** | Doctor sources | Within institution | Within interfaced hospitals | Participating hospitals within the region |
|  | Daily average of interrogation | 50*** | 400*** | Not Available |
|  | Resource specificity | Relatively high | Relatively low | Relatively high |
| **Demanders** | User scale | 620million**** | 298million***** | Over 25million******* |
|  | Application area | Most of provinces and cities in China | All regions in China | Henan, Tianjin, Shandong, Fujin take lead to explore and land the project |
|  | Diagnosis fee | Mainly fees for drugs, fees for diagnosis and treatment are relatively low, fees have been included into medical insurance by public hospitals | Flexible, fees for diagnosis and treatment are relatively high | Relatively low, all  fees are included  into medical  insurance |
| **Sustainability of platform model** | Suppliers’ usage intention | Relatively weak | Relatively strong | Relatively weak |
|  | Demanders’ usage intention | Relatively strong | Very strong | Relatively strong |
|  | Sustainability of platform model | Relatively weak | Relatively strong | Remains to be seen |

Note:

*URL: https://www.cn-healthcare.com/article/20190122/content-513900.html, licensing time: 8June, 2022.

** Statistics as of June 2021, URL: <https://www.cn-healthcare.com/articlewm/20210910/content-1262767.html>, <http://www.199it.com/archives/1522671.html>

*** http://www.199it.com/archives/1522671.html

****URL: <https://www.cn-healthcare.com/article/20200708/content-539141.html>

*****URL: <https://new.qq.com/omn/20220425/20220425A01I9G00.html>

******Statistics as of June 2021，URL: www.cac.gov.cn/2019-10/25/c_1573534818368261.htm, licensing time: 6June, 2022.
